# Facile approach to prepare multi-walled carbon nanotubes/graphene nanoplatelets hybrid materials

**DOI:** 10.1186/1556-276X-8-243

**Published:** 2013-05-16

**Authors:** Yuchen Jia, Kejing Yu, Kun Qian

**Affiliations:** 1 Key Laboratory of Eco-textiles, Ministry of Education, Jiangnan University, Wuxi, Jiangsu 214122, People's Republic of China

**Keywords:** Multi-walled carbon nanotubes, Graphene, Hybrid materials, Poly(acryloyl chloride), Microstructure

## Abstract

A facile approach was developed to prepare multi-walled carbon nanotubes/graphene nanoplatelets hybrid materials through covalent bond formation. First, poly(acryloyl chloride) was grafted onto oxidized multi-walled carbon nanotubes through the reaction between the acyl chloride groups of poly and the hydroxyl groups of oxidized multi-walled carbon nanotubes. Second, the remaining acyl chloride groups of poly were allowed to react with the hydroxyl groups of hydroxylated graphene nanoplatelets. Scanning electron microscopy and transmission electron microscopy data showed that the multi-walled carbon nanotubes and graphene nanoplatelets were effectively connected with each other. And Fourier transform infrared spectroscopy data indicated the formation of covalent bonds between carbon nanotubes and graphene nanoplatelets. Conformational changes were monitored by Raman spectroscopy. This novel kind of carbon hybrid materials may have the potential application in a wide field, especially in increasing the toughness and strength of the matrix resin.

## Background

Recently, hybrid composites have attracted large attention and have received increasing interest in various fields [1-4]. Researchers with different mixtures have been tried out, such as multi-walled carbon nanotubes (MWCNTs) with carbon black [1], few layer graphene with single-walled carbon nanotubes [2], and MWCNTs with graphene nanoplatelets (GnPs) [3]. Kumar et al. [5] brought together hybridized graphitic nanoplatelets and commercially functionalized MWCNTs in a polyetherimide composite. The results revealed a synergistic interaction between the GnPs and MWCNTs based on GnPs protection against fragmentation of the MWCNTs during high-power sonication. Chao Zhang et al. [6] revealed that the graphene oxide (GO) assisted the dispersion of pristine MWCNTs in aqueous media. Moreover, the solubility results indicated that the GO sheets leaned towards stabilizing MWCNTs with larger diameters, mainly depending on whether the MWCNTs are inclined to form bundles, twisted structures, or MWCNTs/GO complexes. S. Chatterjee et al. [4] studied the mechanical reinforcement in a widely used epoxy matrix with the addition of GnPs and various mixture ratios of MWCNTs with GnPs. It had been indicated that the size and synergy effects of nanofiller hybrids including GnPs and MWCNTs played an important role in the mechanical properties of epoxy composites. As mentioned above, these hybrid materials were obtained via the unstable π-stacking interaction, which could be damaged by mechanical stirring or long-time ultrasound. Young-Kwan Kim et al. [7] formed graphene oxide scrolls around MWCNT templates through covalent bond formation. Graphene oxide sheets were successfully made to adopt a scroll conformation around the surface of aminated MWCNT in solution by covalent bond formation. Like the stick wrapped with a film, the microstructure of this kind of hybrid material was still two-dimensional (2D) structure.

In this work, we chose carbon nanotubes and graphene nanoplatelets to prepare three-dimensional (3D)-structured hybrid materials. Due to their unique tubular structure, carbon nanotubes mainly reflect rigidity, while graphene nanoplatelets appear to have better toughness owing to its laminated structure [8-10]. A methodology of preparing multi-walled carbon nanotubes/graphene platelets (MWCNTs/GnPs) hybrid materials was proposed, using poly(acryloyl chloride) as bridges between carbon nanotubes and GnPs. Compared with the other hybrid methods [4-7], this approach is facile, efficient, and easy to control by regulating and controlling polymer chains of poly(acryloyl chloride) (PACl) which can provide numerous reactive groups. In addition, based on the theory of hybrid structure [11], this novel kind of MWCNTs/GnPs hybrid materials can combine the advantages of carbon nanotubes and graphenes, which would make this unique hybrid structures possess the potential application in a wide field, especially in increasing the toughness and strength of the matrix resins. The preparation process involved the following three steps: Firstly, hydroxyl groups on the surface of acid-oxidized multi-walled carbon nanotubes (MWCNTs-OH) reacted with linear PACl to generate highly reactive polymer grafting on the nanotube surface [12,13]. Secondly, the generation of MWCNTs/GnPs hybrid materials could be obtained by the reaction of the acyl chloride groups of PACl on the surface of PACl-grafted MWCNTs (MWCNTs-PACl) and the hydroxyl groups on the surface of hydroxylated GnPs (GnPs-OH). Since PACl provided numerous reactive sites, a large quantity of MWCNTs could be assembled surrounding the GnPs.

## Main text

### Experimental section

#### Materials

MWCNTs-OH (95% pure, length of <5 μm, main range of outer diameter was 20 to 40 nm) were purchased from Shenzhen Nanotech Port Co Ltd. (Shenzhen, China). Graphene nanoplatelets (GnPs) (diameter of 1 to 20 μm, thickness of 5 to 15 nm) were purchased from Xiamen Knano Graphene Technology Co. Ltd. (Xiamen, China). Acryloyl chloride was supplied by J & K Scientific Ltd. (Shanghai, China). Nitric acid, sulfuric acid, tetrahydrofuran (THF), 1,4-dioxane and 2,2′-azosiobutyrontrile (AIBN) were provided by Sinopharm Chemical Reagent Co. Ltd. (Shanghai, China).

#### Preparation of carbon nanotubes/graphene hybrid materials

**Figure 1:**
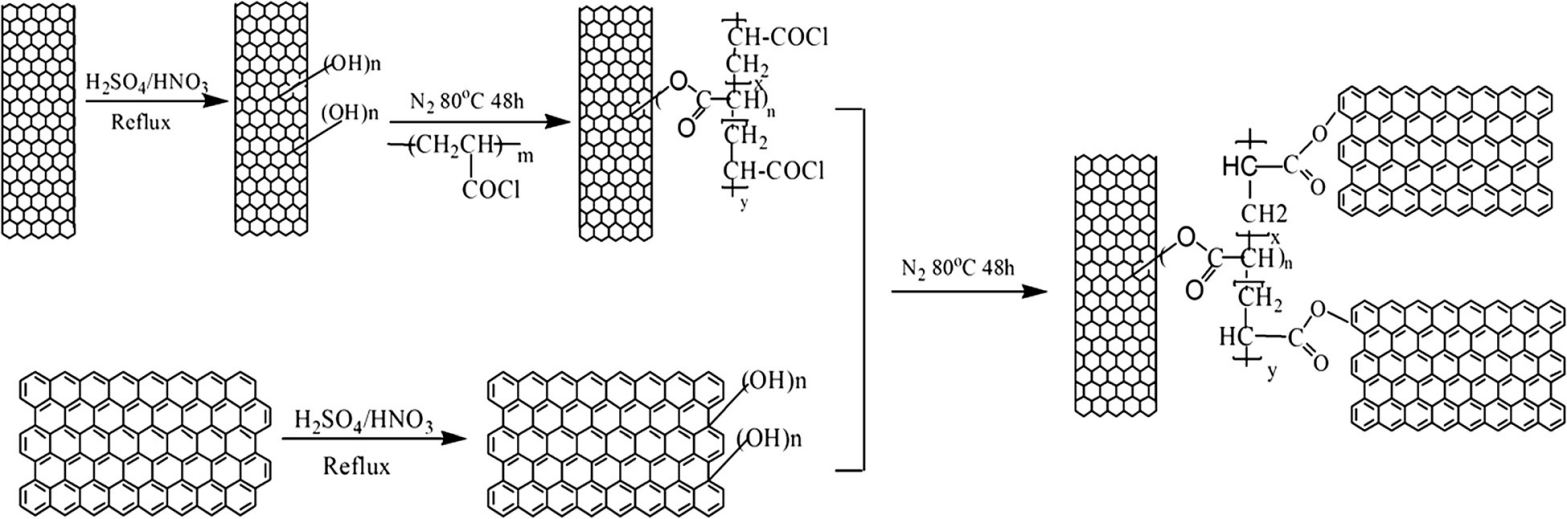
Illustration of the synthesis procedure of MWCNTs/GnPs hybrid materials.

The pristine GnPs were treated with the mixture H_2_SO_4_/HNO_3_ (1:1 *v*/*v*) to obtain the hydroxylated-GnPs (GnPs-OH) [14]. PACl was prepared via free radical polymerization of acryloyl chloride at 60°C in 1,4-dioxane in the presence of AIBN for 48 h in nitrogen atmosphere. The above-obtained PACl was introduced into the suspension of MWCNTs-OH in anhydrous 1,4-dioxane and kept stirred for 48 h under nitrogen atmosphere. MWCNTs-PACl were obtained by collecting after being washed and filtrated repeatedly with THF until pH = 7. Then GnPs-OH were suspended in 1,4-dioxane by ultrasonic dispersion for 4 h. The obtained GnPs-OH suspension and triethylamine were introduced into MWCNTs-PACl suspension and subsequently kept stirred for 48 h at 80°C under nitrogen atmosphere [11]. All the samples of functionalized MWCNTs were soaked in THF for 1 week and then washed repeatedly with THF until pH = 7, followed by drying under vacuum for 12 h at 50°C. The weight of the samples after these processes was almost unchanged, which indicated that the polymer layer was indeed covalently linked to the carbon nanotubes. The synthesis method as described above was presented in Figure 1.

#### Characterizations

The morphologies of the products were observed by scanning electron microscopy (SEM, Hitachi SU1510; Hitachi Ltd. (China), Beijing, China) and transmission electron microscopy (TEM, H-800-1), with the accelerating voltage of 20 to 30 kV, respectively. The microstructures of the samples were analyzed by Fourier transform infrared spectroscope (FTIR, Nexus 670; Thermo Fisher Scientific, Hudson, NH, USA) and Raman spectrometer. Thermal gravimetric analysis (TGA) was conducted on a TGA/SDTA851e instrument at a heating rate of 10°C/min in a nitrogen flow.

## Discussion

### The morphology analysis

**Figure 2:**
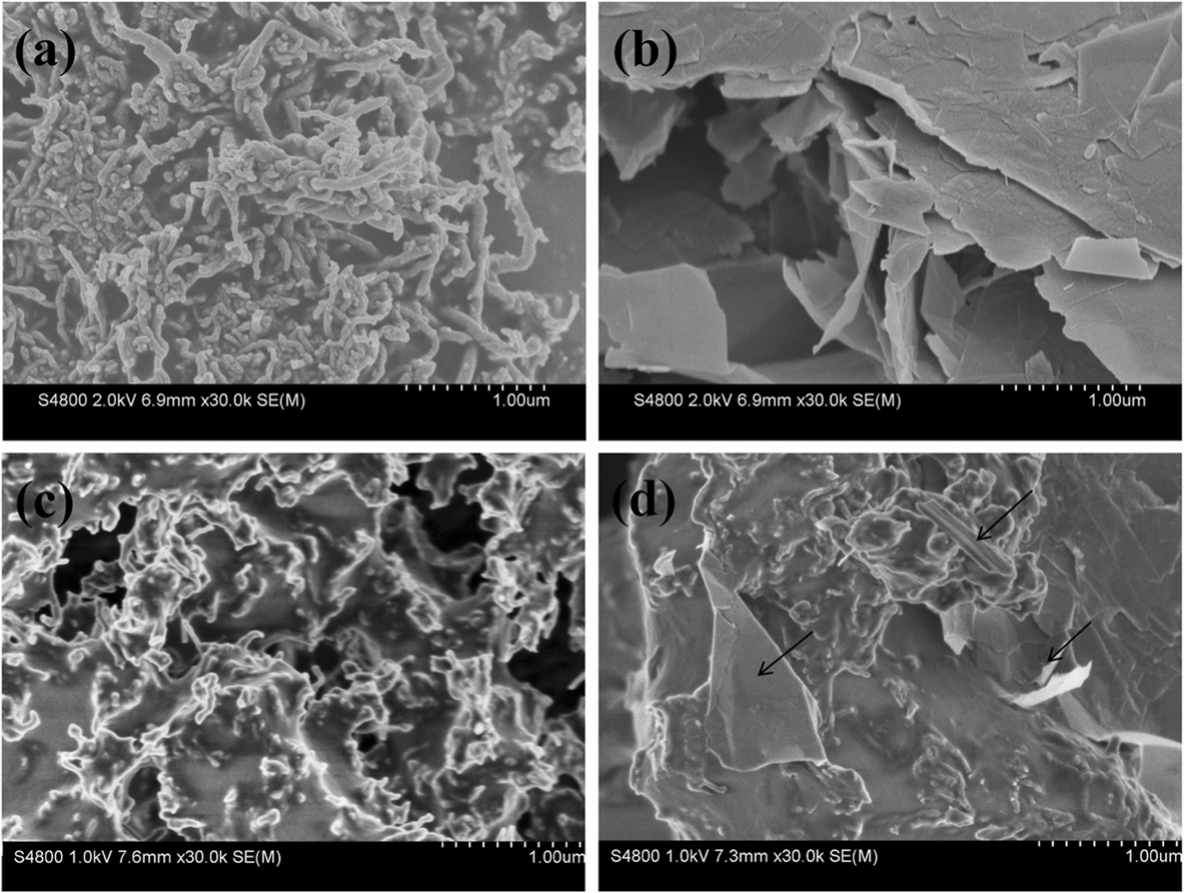
**SEM images.** (**a**) MWCNTs-OH. (**b**) GnPs-OH. (**c**) MWCNTs-PACl. (**d**) MWCNTs/GnPs hybrid materials.

Figure 2 compared the morphology of various nanomaterials. As shown in Figure 2, it could be found that a large quantity of MWCNTs-OH entangled and overlapped into a network structure. Compared to MWCNTs-OH (Figure 2a), well-distributed MWCNTs coated with a layer of polymer could be seen clearly in Figure 2c. For the MWCNTs/GnPs hybrid materials (Figure 2d), both laminated structure of GnPs-OH and tubular structure of MWCNTs could be found. The results indicated that the MWCNTs/GnPs hybrid materials had been synthesized successfully and our chemical grafting method was appropriate.

**Figure 3:**
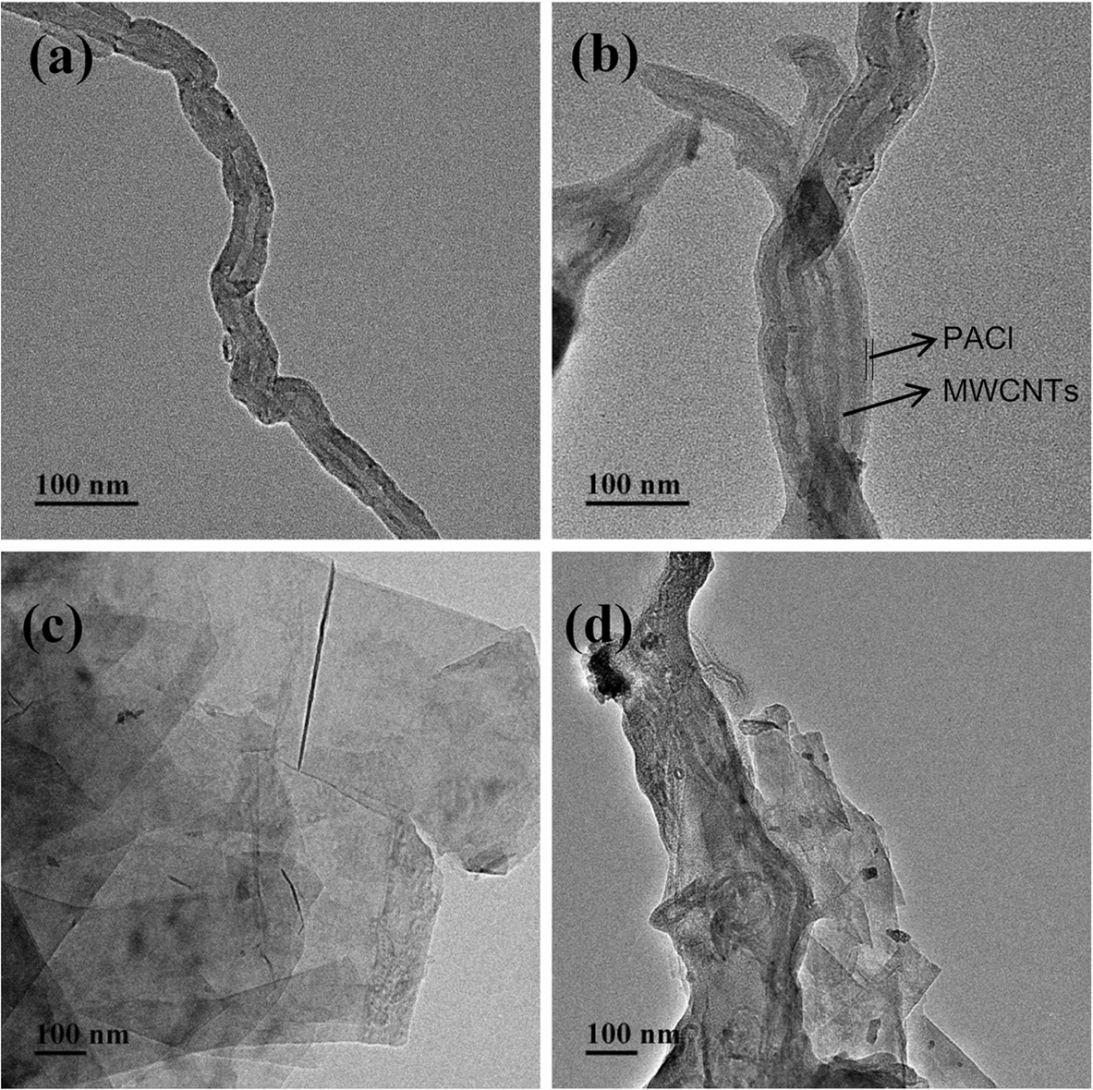
**TEM images.** (**a**) MWCNTs-OH. (**b**) MWCNTs-PACl. (**c**) GnPs-OH. (**d**) MWCNTs/GnPs hybrid materials.

More detailed evidences of microstructure of various MWCNTs nanomaterials could be supported by the TEM images in Figure 3 when compared to the morphology of various nanomaterials. As shown in Figure 3a, the surface of MWCNTs-OH was relatively smooth and clean and exhibited a semitransparent appearance. In contrast, the edge of MWCNTs-PACl (Figure 3b) became substantially thickened with the edge blurred, indicating that the surface of MWCNTs was wrapped by the PACl [11]. It could be seen clearly that the MWCNTs-PACl were hanged on the surface of GnPs (Figure 3d). After those process mentioned above in the ‘Experimental’ section, the weight of samples was almost unchanged which indicated that the polymer layer was indeed covalently linked to the carbon nanotubes. Therefore, it could be confirmed that MWCNTs were assembled onto the surface of GnPs through the reaction of the hydroxyl groups of GnPs and the acyl chloride groups of PACl.

#### The structure analysis

**Figure 4:**
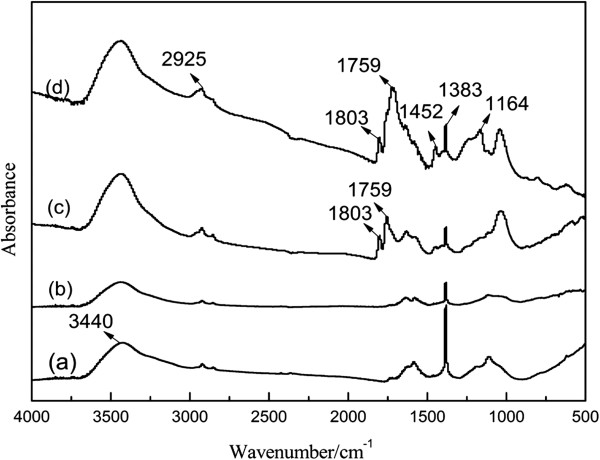
**FTIR spectra.** (**a**) MWCNTs-OH. (**b**) GnPs-OH. (**c**) MWCNTs-PACl. (**d**) MWCNTs/GnPs hybrid materials.

FTIR spectra of various MWCNTs nanomaterials were presented in Figure 4. The C-H stretch vibration of PACl backbone was detected at 2,925 cm^−1^ as a broad and weak absorption peak, while the 1,759 and 1,803 cm^−1^ peaks were originated from characteristic C=O stretching vibration of ester and acyl chloride respectively [14,15]. The FTIR feature in Figure 4c suggested that the PACl was attached to the surface of MWCNTs. Figure 4b showed the features of GnPs: a broad hydroxyl group-related absorption band (3,440 cm^−1^). In Figure 4c and d, the peak of 1,759 cm^−1^ was attributed to the C=O stretching vibrations of the ester carbonyl group, which resulted from the reaction between MWCNTs-PACl and GnPs. In addition, the appearance of an intense absorption peak (C-O, 1,164cm^−1^) indicated the formation of ester linkage between GnPs and MWCNTs-PACl.

**Figure 5:**
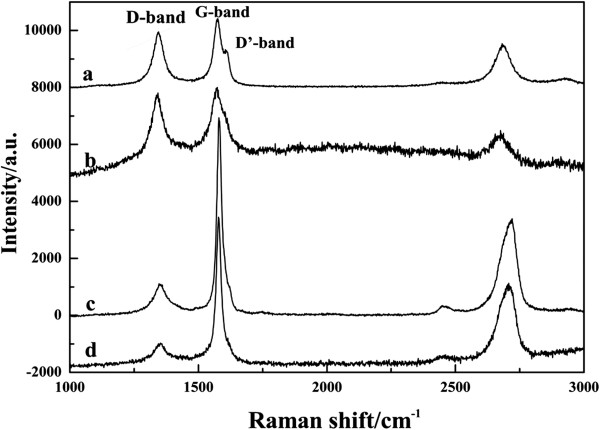
**Raman spectra images.** (**a**) MWCNTs-OH. (**b**) MWCNTs-PACl. (**c**) GnPs-OH. (**d**) MWCNTs/GnPs hybrid materials.

Figure 5 showed the Raman spectra of the samples. All spectra were excited with visible (532 nm) laser light. Raman spectroscopy is a powerful tool in investigating the crystalline, nanocrystalline, and amorphous structures of graphitic-based materials [16,17]. The D band at approximately 1,330 cm^−1^ is attributed to the defects in the disorder-induced modes (or sp3-hybridized carbons), which becomes active in the presence of disorder. The G band at approximately 1,580 cm^−1^ is usually attributed to the in-plane bond-stretching motion of the pairs of C(sp^2^) atoms [3]. Thus, the area ratio of D band to G band (ID/IG) indicates that structure quality. It was obvious that the MWCNTs/GnPs hybrid materials had the lowest ratio (0.3051) compared to MWCNTs-OH (0.8435), MWCNT-PACl (0.7254), and GnPs-OH (0.3653). The change on the ratio of ID/IG meant that a higher defect level was caused by the grafting the polymer chain onto the wide surface area of graphene as well as to the passivation of dangling bonds onto the surface of the MWCNTs [18].

In addition, it should be noted that the G band of MWCNTs was divided into two bands, and the new D′ band at 1,604 cm^−1^ could be related to the extent of the disorder [19,20]. It was worth noting that the D′ band was hardly observed for other samples, which indicated that GnPs and hybrid materials have the smallest amount of impurities. Consequently, the hybrid materials possess higher mechanical properties and thermal conductivity with high crystalline structures [11,21].

#### Thermal gravimetric analysis

**Figure 6:**
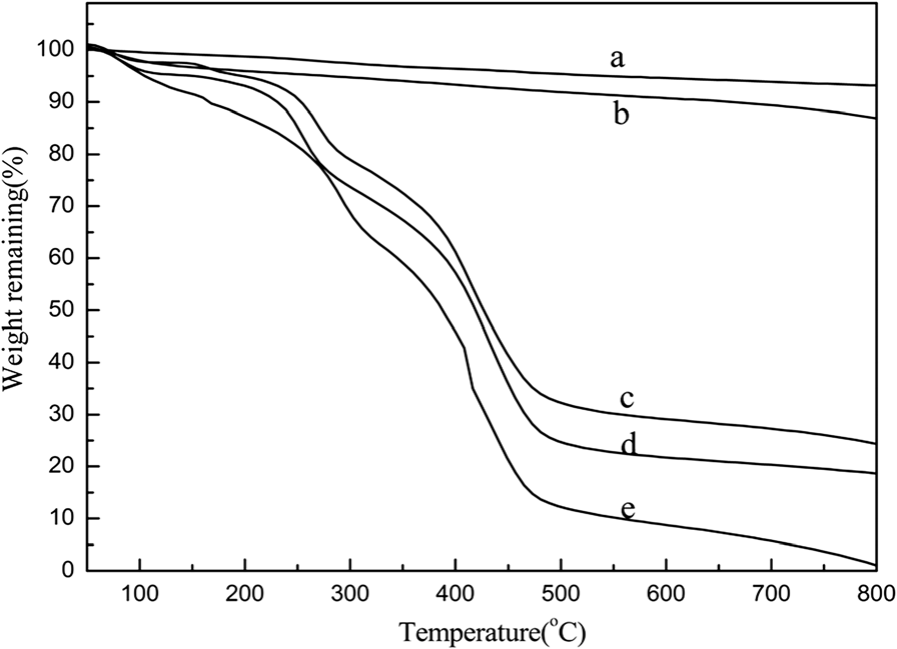
**TG curves.** (**a**) GnPs-OH. (**b**) MWCNTs-OH. (**c**) MWCNTs/GnPs hybrid materials. (**d**) MWCNTs-PACl. (**e**) PACl.

Figure 6 showed the thermogravimetric curves for various samples. The weight-loss behavior of MWCNTs/GnPs (Figure 6c) and MWCNTs-PACl (Figure 6d) could be explained in comparison with those of GnPs-OH (Figure 6a), MWCNTs-OH (Figure 6b), and PACl (Figure 6e). Under N_2_ atmosphere, the GnPs-OH (Figure 6a) and MWCNTs-OH (Figure 6b) showed a slight weight loss owing to the removal of the hydroxyl groups generated by the acid treatment [13]. Neat PACl (Figure 6e) lost about 97% of its original weight before 600°C, and there were two identified stages. The weight loss between 200°C and 300°C was assigned to the decomposition of the side groups of PACl, and the weight loss between 320°C and 550°C was likely due to the decomposition of the polymer backbone. Similarly, the curves for MWCNTs-PACl (Figure 6d) and MWCNTs/GnPs hybrid materials (Figure 6c) almost coincided with the curves of the neat PACl underwent a two-stage weight reduction in the same temperature regions. As shown in Figure 6c, besides the weight loss of PACl occurring at about 400°C, the initial weight loss after 500°C resulted from the presence of GnPs-OH. By referring to the formula in [22], we calculated that the residual weight fraction of polymer on MWCNTs-PACl was about 72% and that of GN-OH on hybrid was about 11% at 600°C. From characterization results of TGA, TEM, and SEM, the covalent linkage of PACl molecules on the surface of MWCNTs and GnPs was confirmed [23].

## Conclusions

In summary, MWCNTs/GnPs hybrid materials can be successfully obtained by a facile method using PACl as a bridge. MWCNTs were assembled onto the surface of GnPs through the reaction of the hydroxyl groups of GnPs and the acyl chloride groups of PACl. The results showed the unique microstructure and excellent thermal properties of MWCNTs/GnPs hybrid materials. It is a pleasure to see that MWCNTs/GnPs hybrid materials make their respective advantages complementary to each other as designed. Therefore, the presented approach will show a potential for preparing carbon hybrid materials through using polymer chains as bridges.

## Abbreviations

FTIR: Fourier transform infrared spectroscope; GnPs: graphene nanoplatelets; GnPs-OH: hydroxylated graphene nanoplatelets; GO: graphene oxide; MWCNTs: multi-walled carbon nanotubes; MWCNTs/GnPs: multi-walled carbon nanotubes/graphene nanoplatelets hybrid materials; PACl: poly(acryloyl chloride); MWCNTs-OH: oxidized multi-walled carbon nanotubes; SEM: scanning electron microscopy; TEM: Transmission electron microscopy; TGA: Thermal gravimetric analysis

## Competing interests

The authors declare that they have no competing interests.

## Authors' contributions

KY and KQ gave the guidance, and YJ did the experiments. KY and YJ analyzed the data and gave the final approval of the version of the manuscript to be published. All authors read and approved the final manuscript.

## References

[B1] Sumfleth J Adroher X Schulte K Synergistic effects in network formation and electrical properties of hybrid epoxy nanocomposites containing multi-wall carbon nanotubes and carbon black J Mater Sci 2009 8 3241 3247 10.1007/s10853-009-3434-7

[B2] Prasad KE Das B Maitra U Ramamurty U Rao C Extraordinary synergy in the mechanical properties of polymer matrix composites reinforced with 2 nanocarbons Proc Natl Acad Sci 2009 8 13186 13189 10.1073/pnas.0905844106 19651605 PMC2726345

[B3] Yang SY Lin WN Huang YL Tien HW Wang JY Ma CC Li SM Wang YS Synergetic effects of graphene platelets and carbon nanotubes on the mechanical and thermal properties of epoxy composites Carbon 2011 8 793 803 10.1016/j.carbon.2010.10.014

[B4] Chatterjee S Nafezarefi F Tai NH Schlagenhauf L Nüesch FA Chu BT Size and synergy effects of nanofiller hybrids including graphene nanoplatelets and carbon nanotubes in mechanical properties of epoxy composites Carbon 2012 8 5380 5386 10.1016/j.carbon.2012.07.021

[B5] Kumar S Sun L Caceres S Li B Wood W Perugini A Maguire RG Zhong WH Dynamic synergy of graphitic nanoplatelets and multiwalled carbon nanotubes in polyetherimide nanocomposites Nanotechnology 2010 8 105702 105711 10.1088/0957-4484/21/10/105702 20154373

[B6] Zhang C Ren LL Wang XY Graphene oxide-assisted dispersion of pristine multiwalled carbon nanotubes in aqueous media J Phys Chem C 2010 8 11435 11440 10.1021/jp103745g

[B7] Kim YK Min DH Preparation of scrolled graphene oxides with multi-walled carbon nanotube templates Carbon 2010 8 4283 4288 10.1016/j.carbon.2010.07.039

[B8] Thostenson ET Ren Z Chou TW Advances in the science and technology of carbon nanotubes and their composites: a review Compos Sci Technol 2001 8 1899 912 10.1016/S0266-3538(01)00094-X

[B9] Gomez-Navarro C Burghard M Kern K Elastic properties of chemically derived single graphene sheet Nano Lett 2008 8 2045 2049 10.1021/nl801384y 18540659

[B10] Park SJ Lee KS Bozoklu G Cai WW Nguyen ST Ruoff RS Graphene oxide papers modified by divalent ions-enhancing mechanical properties via chemical cross-linking ACS Nano 2008 8 572 578 10.1021/nn700349a 19206584

[B11] Liu YX Zhang C Du ZJ Li CJ Li Y Li H Yang XP The preparation of multi-walled carbon nanotubes encapsulated by poly(3-acrylaminopropylsiloxane) with silica nanospheres on the polymer surface Carbon 2008 8 1670 1677 10.1016/j.carbon.2008.07.017

[B12] Li WL Formation mechanism research of multi-scale and multi-dimension hybrid structures based on self-assembly CNTs. PhD thesis 2011 Northwest University, Optics Department

[B13] Liu YX Du ZJ Li Y Zhang C Li CJ Yang XP Li HQ Surface covalent encapsulation of multiwalled carbon nanotubes with poly(acryloyl chloride) grafted poly(ethylene glycol) J Polym Sci Pol Chem 2006 8 6880 6887 10.1002/pola.21748

[B14] Wei W Zhang C Du ZJ Liu YX Li HQ Assembly of fullerenol particles on carbon nanotubes through poly(acryloyl chloride) Mater Lett 2008 8 4167 4169 10.1016/j.matlet.2008.06.005

[B15] Yang YS Qi GR Qian JW Yang SL Acryloyl chloride polymer Appl Polym Sci 1998 8 665 670 10.1002/(SICI)1097-4628(19980425)68:4<665::AID-APP18>3.0.CO;2-Q

[B16] Ferrari AC Robertson J Interpretation of Raman spectra of disordered and amorphous carbon Phys Rev B 2000 8 14095 14107 10.1103/PhysRevB.61.14095

[B17] Ferrari AC Meyer JC Scardaci V Casiraghi C Lazzeri M Mauri F Raman spectrum of graphene and graphene layers Phys Rev Lett 2006 8 187401 187404 17155573 10.1103/PhysRevLett.97.187401

[B18] Patole AS Patole SP Jung SY Yoo JB An JH Kim TH Self assembled graphene/carbon nanotube/polystyrene hybrid nanocomposite by *in situ* microemulsion polymerization Eur Polym J 2012 8 252 259 10.1016/j.eurpolymj.2011.11.005

[B19] Zhang Y Broekhuis AA Stuart MCA Landaluce TF Fausti D Rudolf P Cross-linking of multiwalled carbon nanotubes with polymeric amines Macromolecules 2008 8 6141 6146 10.1021/ma800869w

[B20] Yang Y Xie X Wu J Yang Z Wang X Mai YW Multiwalled carbon nanotubes functionalized by hyperbranched poly(urea-urethane)s by a one-pot polycondensation Macromol Rapid Commun 2006 8 1695 1701 10.1002/marc.200600413

[B21] Jorio A Pimenta MA Souza Filho AG Saito R Dresselhaus G Dresselhaus MS Characterizing carbon nanotube samples with resonance Raman scattering New J Phys 2003 8 139 1–17

[B22] Zhou HF Zhang C Li HQ Du ZJ Fabrication of silica nanoparticles on the surface of functionalized multi-walled carbon nanotubes Carbon 2011 8 126 132 10.1016/j.carbon.2010.08.051

[B23] Zhu YL Du ZJ Li HQ Zhang C Preparation and crystallization behavior of multiwalled carbon nanotubes/poly(vinyl alcohol) nanocomposites Polymer Eng Sci 2011 8 1770 1779 10.1002/pen.21964

